# Supplementary material for: Causal Relationship between Aging and Anorexia Nervosa: A White-Matter-Microstructure-Mediated Mendelian Randomization Analysis
Source: Biomedicines. 2024 Aug 16;12(8):1874. doi: 10.3390/biomedicines12081874 (PMC11351342; doi:10.3390/biomedicines12081874)
Supplement: Supplementary file 1 [file biomedicines-12-01874-s001.zip › Supplementary Figure/Figure S4/Singe SNP plot of MR analysis for TL to ubm-b-1959.pdf]

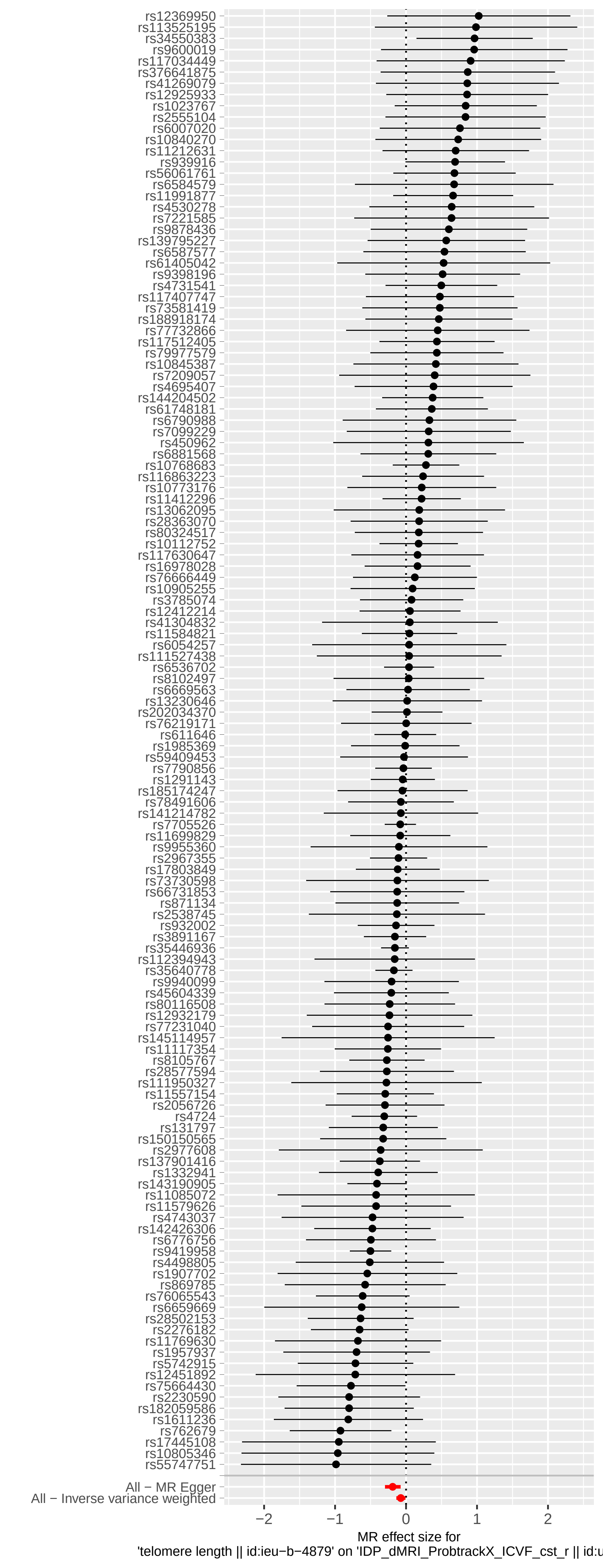
rs12369950
rs113525195
rs34550383
rs9600019
rs117034449
rs376641875
rs41269079
rs12925933
rs1023767
rs2555104
rs6007020
rs10840270
rs11212631
rs939916
rs56061761
rs6584579
rs11991877
rs4530278
rs7221585
rs9878436
rs139795227
rs6587577
rs61405042
rs9398196
rs4731541
rs117407747
rs73581419
rs188918174
rs77732866
rs117512405
rs79977579
rs10845387
rs7209057
rs4695407
rs144204502
rs61748181
rs6790988
rs7099229
rs450962
rs6881568
rs10768683
rs116863223
rs10773176
rs11412296
rs13062095
rs28363070
rs80324517
rs10112752
rs117630647
rs16978028
rs76666449
rs10905255
rs3785074
rs12412214
rs41304832
rs11584821
rs6054257
rs111527438
rs6536702
rs8102497
rs6669563
rs13230646
rs202034370
rs76219171
rs611646
rs1985369
rs59409453
rs7790856
rs1291143
rs185174247
rs78491606
rs141214782
rs7705526
rs11699829
rs9955360
rs2967355
rs17803849
rs73730598
rs66731853
rs871134
rs2538745
rs932002
rs3891167
rs35446936
rs112394943
rs35640778
rs9940099
rs45604339
rs80116508
rs12932179
rs77231040
rs145114957
rs11117354
rs8105767
rs28577594
rs111950327
rs11557154
rs2056726
rs4724
rs131797
rs150150565
rs2977608
rs137901416
rs1332941
rs143190905
rs11085072
rs11579626
rs4743037
rs142426306
rs6776756
rs9419958
rs4498805
rs1907702
rs869785
rs76065543
rs6659669
rs28502153
rs2276182
rs11769630
rs1957937
rs5742915
rs12451892
rs75664430
rs2230590
rs182059586
rs1611236
rs762679
rs17445108
rs10805346
rs55747751
All − MR Egger
All − Inverse variance weighted
−2
−1
0
1
2
MR effect size for
'telomere length || id:ieu−b−4879' on 'IDP_dMRI_ProbtrackX_ICVF_cst_r || id:u
